# A DNA Repair BRCA1 Estrogen Receptor and Targeted Therapy in Breast Cancer

**DOI:** 10.3390/ijms131114898

**Published:** 2012-11-14

**Authors:** Adisorn Ratanaphan

**Affiliations:** Laboratory of Pharmaceutical Biotechnology, Department of Pharmaceutical Chemistry, Faculty of Pharmaceutical Sciences, Prince of Songkla University, Hat-Yai, Songkhla 90112, Thailand; E-Mail: adisorn.r@psu.ac.th; Tel.: +66-7428-8867; Fax: +66-7442-8239

**Keywords:** BRCA1, estrogen receptor, fulvestrant, antiestrogen, triple-negative breast cancer

## Abstract

BRCA1 is a key mediator of DNA repair pathways and participates in the maintenance of the genomic integrity of cells. The control of DNA damage repair mechanisms by BRCA1 is of great interest since molecular defects in this pathway may reflect a predictive value in terms of a cell’s sensitivity to DNA damaging agents or anticancer drugs. BRCA1 has been found to exhibit a hormone-dependent pattern of expression in breast cells. Wild-type BRCA1 is required for the inhibition of the growth of breast tumor cells in response to the pure steroidal ERα antagonist fulvestrant. Also a loss of BRCA1-mediated transcriptional activation of ERα expression results in increased resistance to ERα antagonists. Platinum-based drugs, poly(ADP-ribose) polymerase (PARP) inhibitors, and their combination are currently included in chemotherapy regimens for breast cancer. Preclinical and clinical studies in a *BRCA1*-defective setting have recently indicated a rationale for the use of these compounds against hereditary breast cancers. Initial findings indicate that neoadjuvant use of cisplatin results in high rates of complete pathological response in patients with breast cancer who have BRCA1 mutations. Cisplatin produces a better response in triple-negative breast cancer (TNBC) than in non-TNBC diseases in both the neoadjuvant and adjuvant settings. This implies that TNBC cells may harbor a dysfunctional BRCA1 repair pathway.

## 1. *Breast Cancer Suppressor Gene 1 (BRCA1)* and its Encoded Protein

**Figure 1:**
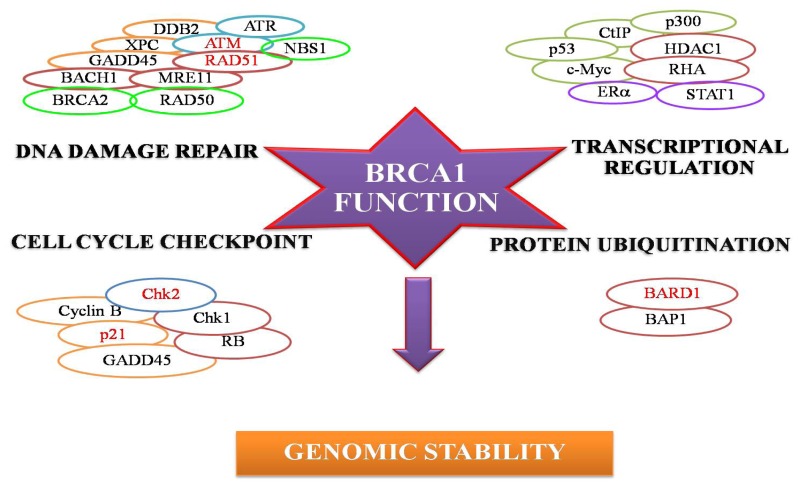
BRCA1 interacting proteins.

*BRCA1* is a tumor suppressor gene consisting of 5592 base pairs spanning 24 exons; 22 exons of which encode a 220 kDa protein of 1863 amino acids together with two non-coding exons. The encoded BRCA1 is functionally characterized into three major domains including a *N*-terminal RING domain, a nuclear localization signal domain (NLS) and a BRCA1 *C*-terminal domain (BRCT domain) [1,2]. The BRCA1 protein has multi-functions in at least four major areas of cellular processes, including DNA repair, transcriptional activation, cell cycle regulation, chromatin remodeling and protein ubiquitination (Figure 1).

The *N*-terminal RING domain contains the conservative sequences of cysteine and histidine residues necessary for specific coordination with two Zn^2+^ ions. This region of BRCA1 interacts with a BARD1 (BRCA1 associated RING domain 1) to form a heterodimeric complex. The BRCA1-BARD1 complex requires both parts for their mutual stability. They are co-localized in nuclear dots during the S phase but not the G phase of the cell cycle as well as in nuclear foci [3]. The progression to the S phase by aggregation of nuclear BRCA1 and BARD1 implied the importance of both proteins for a DNA repair function [4]. The BRCA1-BARD1 complex also exhibits enzymatic activity of an E3 ubiquitin ligase that specifically transfers ubiquitin to protein substrates which are essential for cellular viability [3,5]. The central region of BRCA1 also called the nuclear localization signal domain, covers exon 11 (approximately 3500 bp) and constitutes approximately 60 percent of the coding region of the gene. Deletion of exon 11 results in removal of the nuclear localization signal of BRCA1. Biophysical characterization revealed that this domain is intrinsically disordered or is negatively unfolded under physiological conditions. This might potentially allow the BRCA1 central region to act as a long flexible scaffold, to mediate interactions with DNA, and perhaps a number of other proteins involved in DNA damage response and repair [6]. The reported binding partner proteins to the central region are c-Myc, RB, p53, FANCA, RAD50, RAD51, and BRCA2 [7]. The *C*-terminal domain of BRCA1 (residues 1646–1863) contains two BRCT (BRCA1 *C*-terminal) domains in tandem (motif 1: amino acids 1653–1736); motif 2: amino acids 1760–1855). These domains serve as multipurpose protein-protein interaction modules that bind to other BRCT repeats or other protein domains with apparently unrelated structures [8].

Since all these processes are involved in the maintenance of genomic stability, BRCA1 has been implicated as a key regulator of cellular response to DNA damage [9,10]. One important function of BRCA1 is the repair of DNA damage. The link between BRCA1 and DNA repair has demonstrated that BRCA1 colocalizes with a homologous recombinase, RAD51 [11]. BRCA1 is implicated in playing a crucial role in DNA repair through several mechanisms, including homologous recombination (HR) repair, the less error-prone mechanism of repairing DNA double-strand breaks (DSBs) [12]. Induction of DSB, the most destructive and cytotoxic DNA lesion, by irradiation or anticancer agents is a major strategy employed for breast cancer treatment. To repair the lesions, cells perform a DNA-damage response that includes chromatin remodeling, activation of cell-cycle checkpoints, DNA repair and, allowing time for the DNA repair to occur. If the responses fail, cells undergo apoptosis as a last resort to sustain genomic stability. DSB preferentially causes breast cancer cells to undergo apoptosis, especially when relevant repair pathways, such as those mediated by BRCA1, are perturbed [13]. Several lines of evidence have indicated that inactivation of the genes required for a DNA double-strand break (DSB) repair pathway including *ATM*, *MDC*, *BRCA1*, *BRCA2*, and *RAD51*, causes cells to become hypersensitive to DSB-inducing agents.

**Figure 2:**
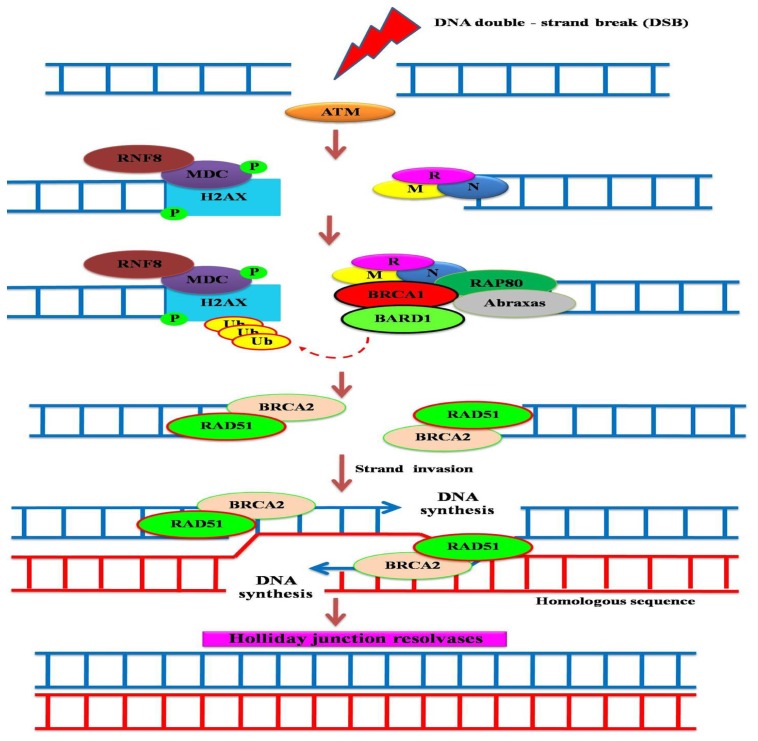
BRCA1-mediated homologous recombination (HR) repair. DNA double-strand break (the most destructive and cytotoxic DNA lesion is induced by irradiation or anticancer agents) activates the protein kinase ATM. The MRE11-RAD50-NSB1 (MRN) complex acts as a DSB sensor, recognizes DSB, and recruits ATM to the site of the DNA damage. ATM phophorylates the histone variant H2AX (γ-H2AX) that can directly recruit MDC1. ATM further phosphorylates MDC1, then recruits an E3 ubiquitin ligase, RNF8, that catalyzes polyubiquitin chains at the sites of DNA damage. The ubiquitin polymer next recruits the BRCA1-Abraxas-RAP80 complex through the RAP80 component. BRCA1 forms RING heterodimer E3 ligase activity with BARD1, which is required for recruitment of BRCA2 and RAD51 to damaged sites for HR repair through sister chromatid exchange. Resolvases restore Holliday junctions, and error-free DNA molecules are finally produced.

The molecular mechanism of the HR repair pathway after DSB has recently been dramatically revealed (Figure 2). The Mre11-Rad50-Nbs1 (MRN) complex that acts as a DSB sensor, first recognizes DSB and recruits ATM, a PI3 kinase, that is frequently mutated in ataxia telangiectasia patients, to the site of DNA damage. ATM phophorylates the histone variant H2AX (γ-H2AX) [14,15] which can now directly recruit MDC1. ATM further phosphorylates MDC1 [16–18], then recruits an E3 ubiquitin ligase, RNF8, that catalyzes lysine 63, K63-linked polyubiquitin chains at the sites of DNA damage [19–22]. The K63-linked ubiquitin polymer next recruits the BRCA1-Abraxas-RAP80 complex through the RAP80 component, a protein that contains two UIM (ubiquitin interacting motif) domains [23–25]. BRCA1 can form RING heterodimer E3 ligase activity with BARD1, and this is required for the recruitment of BRCA2 and RAD51 to the damaged sites to initiate HR repair through a sister chromatid exchange [26–28]. Many cancer-predisposing mutations in the BRCA1 RING domain, that inhibited E3 ligase activity and its ability to accumulate at a damaged site, were defective in homologous recombination, which is critical for tumor suppression [27,29,30].

The significance of the DNA repair function of BRCA1 through HR was observed from experiments that showed that BRCA1-deficient mouse embryonic stem cells displayed defective homologous repair of chromosomal recombination with increased frequency of non-homologous recombination. This impairment could be corrected by the reconstitution of a wild-type BRCA1 [31]. Antisense or siRNA-based inhibition of endogenous BRCA1 expression promoted increased sensitivity to cisplatin that was associated with decreased DNA repair and increased apoptosis [32–34]. This indicated that the reduced BRCA1 expression observed in sporadic cancers may also be exploited for DNA damage-based chemotherapy [35,36]. Expression of BRCA1 was found to be about two-fold lower in sporadic triple-negative breast cancers compared to estrogen receptor (ER)-positive cancers [37]. In addition, upregulation of microRNA (miR-182) that targets BRCA1 expression may also lead to BRCA1 pathway dysfunction, and subsequently reduce the efficiency of HR repair [38]. BRCA1-defective human breast cancer, HCC1937 cells (a BRCA1 mutation with an insertion of C at nucleotide 5382, and a negative expression of HER2/neu, and estrogen receptor α (ERα)) were significantly more sensitive to cisplatin than BRCA1 reconstituted cells with a full-length cDNA transfection [39]. In a similar situation, BRCA1-deficient mouse embryonic stem cells displayed defective DNA repair and a 100-fold increased sensitivity to the alkylating agent mitomycin C and cisplatin compared to those containing wild-type BRCA1 [40,41]. This sensitivity was reversed upon correction of the BRCA1 mutation in mouse embryonic fibroblast cells with disrupted BRCA1 [42]. Reconstitution of BRCA1 in the cells via transfection meant that BRCA1 functions were regained, and resulted in a reduced level of cancer cell death, following treatment with cisplatin or other DNA damaging agents [34].

As described earlier, BRCA1 contains a *C*-terminal transactivation domain [43–46]. The transactivation domain is mapped to the region of the protein encoded by exon 21–24 using deletion constructs of BRCA1 fused to the GAL4 DNA binding domain. The BRCA1-BRCT domain has been implicated in the regulation of transcription of several genes responsible for DNA damage. The ability of BRCA1 to act as either a co-activator or a co-repressor of transcription may involve its ability to recruit basal transcription machinery and other proteins that have been implicated in chromatin remodeling [47]. BRCA1 was shown to interact with the RNA polymerase II holoenzyme [11], and is capable of activating the p21 promoter [48]. It has been reported that BRCA1 participated in stabilizing p53 in response to DNA damage, and served as a co-activator for p53 [49]. The interaction of BRCA1 and p53 potentially resulted in the redirection of a p53-mediated transactivation from apoptotic target genes involved in DNA repair and cell cycle arrest [49]. In addition, it has been reported that Smad3, a component of the transforming growth factor β (TGF-β) signaling pathway, which is a potent regulator of growth and apoptosis, also for invasiveness of tumor cells, forms a complex with BRCA1 *in vitro* and *in vivo*. The interaction is mediated by the MH1 domain of Smad3 and the *C*-terminal part of BRCA1. However, Smad3 counteracted the BRCA1-dependent repair of DNA double-strand breaks in human breast epithelial cells, as evaluated by formation of BRCA1 nuclear foci. Smad3, therefore, suppresses BRCA1-dependent DNA repair in response to a DNA damaging agent [50]. In addition, BRCA1 was shown to repress the transcription of ERα and its downstream estrogen responsive genes [51]. The transcriptional repression activity of BRCA1 was found to abolish its ability to inhibit ERα activity that occurs by the association of the *N*-terminus of BRCA1 (residues 1–300) with the *C*-terminal activation function (AF-2) of ERα. As described above, BRCA1 and BARD1 interact through their RING domain to form a heterodimer with E3 ubiquitin ligase activity. The BRCA1 E3 ligase activity has been found to be inactivated by a breast cancer-derived mutation and platinum-based drugs [3,52]. In addition, cancer-predisposing mutations in BRCA1 have been observed to abrogate ERα ubiquitination [53].

## 2. Estrogen, Estrogen Receptor and Breast Cancer

**Figure 3:**
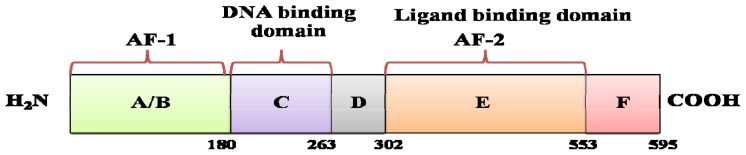
The functional domain of ERα.

Estrogen (E_2_) is important in women for a variety of physiological processes. It affects growth, differentiation, and the function of tissues in the reproductive system, including the mammary glands, uterus, vagina, and ovaries [54,55]. Estrogen action is primarily mediated through binding with nuclear proteins called estrogen receptors (ER). Estrogen receptors are members of nuclear hormone receptors, a family of hormone activated transcription factors that can initiate or enhance the transcription of genes containing specific hormone response elements [56,57]. The ERα protein is encoded from the estrogen receptor 1 gene (*ESR1* gene). It consists of 595 amino acids with a molecular weight of 66 kDa that has been separated into six different functional domains (A–F) (Figure 3) [58,59].

The *N*-terminal A/B domain is involved in activation of ligand-dependent transcription. The DNA binding-domain (DBD) on the *C*-region mediates ERα binding to specific DNA enhancer sequences, called the estrogen responsive element (ERE) with the consensus sequence of 5′-GGTCANNNTGACC-3′ [60,61]. Several three-dimensional structures are known for ERα DBD alone and in complex with DNA [55,61–63]. The topology of ERα DBD is characterized by a zinc finger-like motif with eight cyteines that constitute the tetrahedral coordination of the two zinc ions. The E-region is the ligand-binding domain (LBD) which is responsible for the high affinity for binding of estrogen. The DBD and LBD are connected by the hinge region (D-domain). The F-domain is the *C*-terminal extension region of LBD [64].

**Figure 4:**
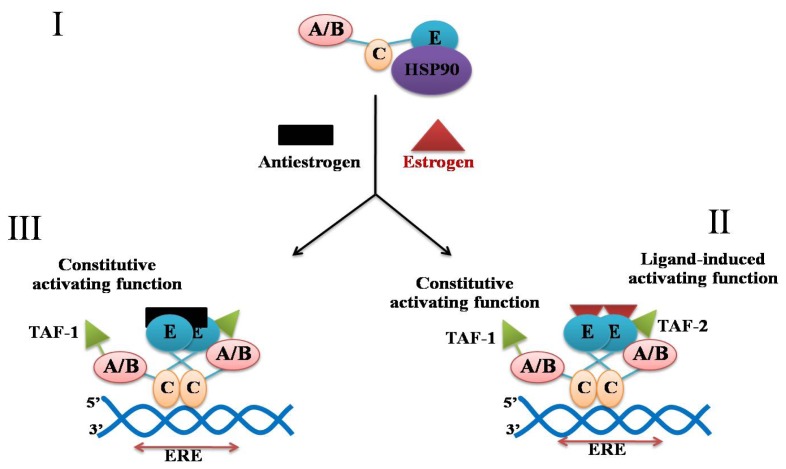
The model for the mechanism of action of estrogen. An unoccupied estrogen receptor (ER) binds to HSP90 (I). Estrogen binds to the region E ligand binding domain (LBD). Antiestrogen tamoxifen is capable of inducing dimerization and DNA-binding, but does not activate the transcription-activation function 2 (TAF-2). ERE is an estrogen responsive DNA element.

The mechanism of estrogen action via ERα is a very complex process (Figure 4). In the non-stimulated receptor, the heat shock protein 90 (HSP90) is positioned in such a way that prevents dimerization and strong binding to the ERE in DNA. The addition of estrogen (E2) is followed by the separation of HSP90, which is accompanied by dimerization, with a strong interaction with the LBD, and a weaker interaction with the DBD. The resulting dimer binds to the ERE and activates transcription through the intervention of the ligand-inducible transcription-activating function 2 (TAF-2: recently called AF2) and the constitutive transcription activation 1 (TAF1: recently called AF1), located at the LBD and the region A/B, respectively [65].

## 3. Association between *BRCA1* Expression and Response to Antiestrogen Treatment

Breast tumorigenesis and breast cancer progression involves the deregulation or hyper- activation of intracellular signaling proteins that leads to uncontrolled cellular proliferation, invasion and metastasis. The estrogen receptor and transforming growth factor β (TGF-β) signaling pathways especially, change during breast tumorigenesis and breast cancer progression, through the downstream mediator, *Smad3*. Several studies have reported that ERα suppresses the expression of *Smad3* induced by estrogen [66]. Reversal of the suppression of *Smad3* activity by ERα/E_2_ decreased, when induced by the antiestrogen tamoxifen, which indicates that this effect is mediated directly from the ERα activity. These findings are consistent with the reported therapeutic effects of antiestrogens such as tamoxifen, through local boosting of TGF-β signaling [67]. Previous studies have reported that induction of TGF-β in a breast cancer cell line revealed that the response to antiestrogen was confined to ER-positive (MCF-7) cells and not ER-negative (MDA-MB-231) cells. ER-positive (MCF-7) cells responded to antiestrogen, tamoxifen, but ER-negative MDA-MB-231 cells did not [68].

The estrogen receptor status is useful in predicting the benefit obtained from endocrine therapy. It may also help predict which patients benefit from advances in adjuvant chemotherapy [69]. In patients with hormone-sensitive tumors, tamoxifen reduces the risk of recurrence and death. Furthermore, treatment with the aromatase inhibitor alone or consecutively with tamoxifen replaces or further reduces the risk of recurrence in post-menopausal women with estrogen receptor-positive tumors [70]. Endocrine therapy with selective estrogen receptor modulators (SERMs) has been the mainstay of breast cancer prevention trials to date. It is also known that tamoxifen exerts pure antagonism on genes that require only the AF-2 domain for ERα-mediated transcriptional activity. In contrast, in genes for which ERα AF-2 is not required, transcription is then driven only by AF-1 and tamoxifen can function as a partial agonist [71,72].

**Figure 5:**
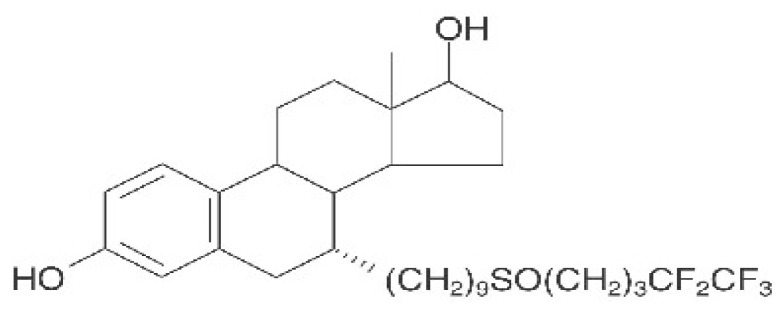
Chemical structure of fulvestrant.

Germline mutations in the *BRCA1* gene confer a genetic predisposition to breast and ovarian cancers. BRCA1-mutant breast cancers exhibit a distinct pathologic phenotype and lack of ERα [73]. BRCA1 has been shown to inhibit ERα signaling, which results in negative regulation of expression of downstream genes [74], as well as regulation of estrogen biosynthesis through transcriptional inhibition of the aromatase encoding genes [75]. Recently, an alternative pathway for breast cancer treatment was described using pure antiestrogen. The effect of BRCA1 expression on the response of breast cancer cells to a pure steroidal ERα antagonist, fulvestrant, has been investigated [76,77] (Figure 5).

**Figure 6:**
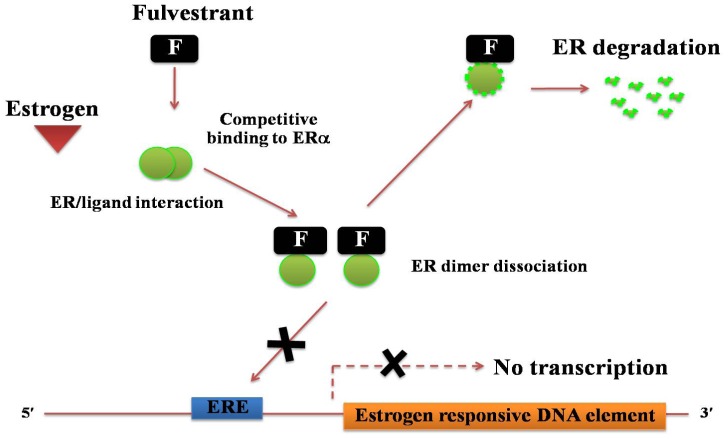
The molecular mechanism for the pure steroidal ERα antagonist, fulvestrant. Fulvestrant binds competitively to ERα with a high affinity. It acts as an antiestrogen chemical by reducing the half-life of ERα, resulting in a decrease in expression of ERα. Formation of the drug-receptor complex leads to stabilization of the receptor, which is degraded by an ubiquitin-proteasome complex.

Unlike the selective estrogen receptor modulator tamoxifen, the primary mechanism of the action of fulvestrant is through downregulation of ERα (Figure 6). Fulvestrant is a steroidal analogue of 17β-estradiol, which competitively binds to ERα with a high affinity [78,79]. It acts as an antiestrogen chemical by reducing the half-life of ERα [80], resulting in a decrease in expression of ERα. Formation of the drug-receptor complex results in stabilization of the receptor, which is then degraded by an ubiquitin-proteasome complex [81–83].

Fulvestrant has been shown to inhibit the growth of cells that were transfected with siRNA [58]. This indicates that the wild-type BRCA1 is required for fulvestrant to inhibit the growth of breast tumor cells, and loss of BRCA1-mediated transcriptional activation of the expression of ERα results in an increased resistance to ERα antagonists [58]. Due to its unique action in the downregulation of the ERα, the mechanism of fulvestrant is described by the acronym “SERD” (Selective ER downregulator). In addition, binding of fulvestrant to the ERα, inhibits ERα dimerization and the uptake of the drug-receptor complex by the nucleus (Figure 6) [79]. Fulvestrant also causes inactivation of two regions of the ERα, the activating function-1 (AF-1) and the activating function-2 (AF-2), that normally recruit coactivator and corepressor proteins required for expression of ERα-regulated genes [80,84]. Considerable data has demonstrated the efficacy of fulvestrant in postmenopausal women with ER-positive advanced breast cancer, both for the first-line setting and following disease progression after tamoxifen or aromatase inhibitors. Recently, fulvestrant was reported to provide improved benefits with alternative dosing strategies. Considering all administration schedules, fulvestrant has an excellent safety profile with no significant adverse effects [85]. More recently, the aromatase inhibitors, that inhibit the final chemical conversion of androgens to estrogens, have shown an increased disease-free survival benefit over tamoxifen in patients with primary hormone receptor-positive breast cancer, as well as reducing the risk of developing contralateral breast cancers [86].

## 4. “Triple-Negative” Breast Cancer and Treatment

Breast cancer is a heterogeneous disease, and gene expression profiling has shown that it is possible to classify and identify five major biologically distinct intrinsic subtypes: luminal A, luminal B, human epidermal growth factor receptor 2 (HER2) overexpression, basal-like, and normal-like [87–89]. These molecular subtypes have prognostic and predictive values as HER2-overexpressing and basal-like breast cancers have poor outcomes. Follow-up studies have shown that these subtypes are conserved across diverse patient series and array platforms [90,91], and have shown that different gene expression-based predictors are a good way for tracking a similar, common set of biological subtypes, with significant agreement in predicting patient outcomes [92]. A subtype of breast cancer, characterized by the lack of expression of estrogen receptor (ER), progesterone (PR), and human epidermal growth factor 2 (HER2) is called a ‘triple-negative’ breast cancer (TNBCs) [93–97]. This subgroup accounts for about 15% of all breast cancers and for a higher percentage of breast cancers detected in African and African-American women who are premenopausal [94]. TNBC has important clinical implications, because it is typicality high grade, has a ductal histology, and exhibits a high rate of proliferation. In general, compared with other subtypes of breast cancer, TNBC has a less favorable clinical outcome in terms of the nature and likelihood of progression, availability of various treatment options, and survival. Although a cure is likely if TNBC is diagnosed early and responds well to treatment, the highly aggressive nature of this disease has contributed to poorer outcomes, overall [95]. TNBC patients are known to have a greater pathological complete response (pCR) rate when compared with non-TNBC patients [98].

Currently, there is no preferred standard form of chemotherapy for TNBC, and treatment should be selected as it is for other cancer subtypes. In the adjuvant setting, anthracyclines and taxanes remain the standard of care for TNBC patients in node-positive breast cancer [99]. Retrospective analysis indicates that the addition of docetaxel or paclitaxel to anthracycline containing adjuvant regimens may be of greater benefit for the treatment of ER-negative and HER2-negative cancers, which are much more common [100,101]. More experimental neoadjuvant regimens including platinum drugs paired with taxane have been shown to achieve high pCR rates in TNBC [98,102]. Newer treatment approaches to the use of platinum agents, cisplatin and carboplatin to treat TNBC are currently being assessed in clinical trials; on the basis that the dysfunction of BRCA1 and its various pathways is associated with a specific DNA-repair defect that sensitizes cells to these agents in an animal model [103]. In addition, single-agent cisplatin induced a response in TNBC patients. Decreased BRCA1 expression in TNBC sensitizes patients to cisplatin [104]. Therefore, cisplatin-based chemotherapy has improved outcomes for treating TNBC patients [105]. The molecular pathway of cisplatin-induced cell death for TNBC has been discovered using triple-negative cells (HCC1937 cells that express only mutationally inactivated BRCA1 and MDA-MB-468 cells that express wild-type BRCA1) [106]. The mediator signal of this pathway is p63/p73. The expression of these genes appears to reflect a functional pathway shared by BRCA1-associated tumors [106]. Inactivation of this pathway increases the IC_50_ of breast cancer cells for cisplatin by 10 to 100 fold. In addition, enhancement of p73 protein expression was also observed in MDA-MB-231, MDA-MB-468 and HCC1937 cells following the treatment of cells with a cisplatin-rapamycin combination [107]. BRCA1 mutant cells that exhibit sensitivity to a gemcitabine and cisplatin combination treatment, appear to be mediated by sustained DNA damage and inefficient DNA repair that triggers p63/p73 mediated apoptosis [108,109]. The p63/p73 proteins are expressed in about 30%–50% of TNBC patients and this might be a biomarker for clinical sensitivity to cisplatin [97,106,110]. Initial findings indicate that neoadjuvant use of cisplatin results in high rates of complete pathological response in patients with breast cancer who have BRCA1 mutations [104,111,112]. This anticancer platinum drug produces a better response in TNBC than in non-TNBC diseases in both the neoadjuvant and adjuvant settings [113].

TNBC tumors are strongly associated with germline mutations in the *BRCA1* gene [114], although much about this relationship remains to be defined [115]. The present data indicates that a defective BRCA1 function could be more specifically targeted by poly (ADP-ribose) polymerase (PARP) inhibitors. PARP has an important role in base excision repair of single-strand DNA breaks. Inhibition of PARP leads to accumulation of single-stranded DNA breaks that can cause the formation of double-stranded DNA breaks, after stalling the progressing DNA replication forks. These double-stranded DNA breaks cannot be accurately repaired in tumors with homologous recombination deficiency [12]. The inhibition of PARP using synthetic killing agents has therefore been advanced as a novel targeted therapy for cancers harboring *BRCA1* mutations [116]. Preclinical data on the mechanisms of PARP inhibitors are at a different stage of clinical development for the targeted treatment of BRCA1-deficient breast cancer and TNBC. PARP inhibitors include olaparib (AZD2281, KU-0059436), iniparib (BSI-201), and veliparib (ABT888). Olaparib has been evaluated in a phase I study. *In vitro* data has shown that inhibition of PARP leads to a highly selective apoptosis of *BRCA1* null cells [117]. The DNA repair defects, that are the characteristics of *BRCA1*-deficient cells, confer sensitivity to PARP inhibition, which could be relevant to the treatment of TNBC [118]. PARP inhibitors have recently shown very encouraging clinical activity in early trials of tumors arising in *BRCA1* mutation [119]. A recent multi-center proof-of-concept phase II trial demonstrated a positive result with a single olaparib treatment in a BRCA1-mutated breast cancer [120]. In addition, olaparib has been tested in combination with cisplatin in TNBC, and with gemcitabine in solid tumors [121,122]. However, iniparib failed to improve survival in TNBC patients in phase III clinical trials in combination with chemotherapy [123]. Nevertheless, it is optimistic that future development of this class of compounds remains necessary to determine the effectiveness of PARP inhibitors in the treatments of breast cancer patients with BRCA1-associated mutations [124].

## 5. Conclusions

The goal of all cancer therapies is to selectively eradicate the cancer while sparing normal tissues. The cellular responses to DNA damage, especially related to repair or tolerance of the damage are critical issues in determining the efficacy of most cancer chemotherapy. The selective sensitivity of cancer cells relative to normal cells should improve the therapeutic ratio for cancer chemotherapy. Cancer cells with defective DNA repair pathways, such as loss of BRCA1, inactivation of the BRCA1 DNA repair pathway, and synthetic lethality, alter their sensitivity to DNA-damaging agents or chemotherapeutic drugs. BRCA1 represses the transcription of ERα and its downstream estrogen responsive genes. Wild-type BRCA1 is required for the inhibition of growth of breast tumor cells in response to the pure steroidal ERα antagonist fulvestrant. Also a loss of BRCA1-mediated transcriptional activation of ERα expression results in increased resistance to ERα antagonists. Platinum-based drugs, poly (ADP-ribose) polymerase (PARP) inhibitors, and their combination are currently included in chemotherapy regimens for breast cancer. Although widely used as anticancer therapeutics, the clinical applications of the anticancer platinum drugs are limited due to their adverse side effects and the cancer cells can also develop resistance to the drugs. Therefore, rationally designed drugs that exert their anticancer activities on both estrogenic activity and synthetic lethality could lead to the discovery of new opportunities for the development of targeted breast cancer therapies.
